# Survey Analysis of Quantitative and Qualitative Menstrual Cycle Tracking Technologies

**DOI:** 10.3390/medicina59091509

**Published:** 2023-08-22

**Authors:** Theresa M. Stujenske, Qiyan Mu, Melisssa Pérez Capotosto, Thomas P. Bouchard

**Affiliations:** 1 Duquesne School of Nursing, Duquesne University, Pittsburgh, PA 15282, USA; 2 Institute for Natural Family Planning, College of Nursing, Marquette University, Milwaukee, WI 53233, USA; qiyan2mu@gmail.com; 3 W. F. Connell School of Nursing, Boston College, Chestnut Hill, MA 02467, USA; 4 Department of Family Medicine, University of Calgary, Calgary, AB T3H 0N9, Canada; tbouchar@ucalgary.ca

**Keywords:** mobile applications, fertility monitoring, luteinizing hormone, urine hormone tests, temperature tracking, ovulation detection

## Abstract

*Background and Objectives*: Digital health and personalized medicine are advancing at an unprecedented pace. Users can document their menstrual cycle data in a variety of ways, including smartphone applications (apps), temperature tracking devices, and at-home urine hormone tests. Understanding the needs and goals of women using menstrual cycle tracking technologies is the first step to making these technologies more evidence based. The purpose of this study was to examine the current use of these technologies and explore how they are being used within the context of common hormonal and reproductive disorders, like polycystic ovary syndrome (PCOS), endometriosis, and infertility. *Materials and Methods*: This was a cross-sectional study evaluating menstrual cycle tracking technology use. Participants were recruited in January–March 2023 using social media groups and a Marquette Method instructor email listserv. Data were collected using an electronic survey with Qualtrics. Data collected included participant demographics, menstrual cycle characteristics, reproductive health history, and menstrual cycle tracking behavior. *Results*: Three-hundred and sixty-eight participants were included in the analysis. Women had various motivations for tracking their menstrual cycles. Most participants (72.8%) selected “to avoid getting pregnant” as the primary motivation. Three hundred and fifty-six participants (96.7%) reported using a fertility awareness-based method to track and interpret their menstrual cycle data. The Marquette Method, which utilizes urine hormone tracking, was the most frequently used method (*n* = 274, 68.2%). The most frequently used cycle technology was a urine hormone test or monitor (*n* = 299, 81.3%), followed by a smartphone app (*n* = 253, 68.8%), and a temperature tracking device (*n* = 116, 31.5%). Women with PCOS (63.6%), endometriosis (61.8%), and infertility (75%) in our study reported that the use of tracking technologies aided in the diagnosis. Most participants (87.2%) reported a high degree of satisfaction with their use and that they contributed to their reproductive health knowledge (73.9%). *Conclusions*: Women in our study reported avoiding pregnancy as their primary motivation for using menstrual cycle tracking technologies, with the most frequently used being a urine hormone test or monitor. Our study results emphasize the need to validate these technologies to support their use for family planning. Given that most women in this study reported using a fertility awareness-based method, the results cannot be generalized to all users of menstrual cycle tracking technologies.

## 1. Introduction

In recent years, there has been tremendous growth in menstrual cycle tracking technologies, aimed at allowing women to easily track their menstrual cycles [1]. The recent advancement in these technologies demonstrates an increasing interest in tracking menstrual cycle data to achieve reproductive health goals [2]. Their expansion also points to a growing awareness of the significance of these data for reproductive health monitoring and management [3]. The rate at which these technologies are developing has made it challenging for health care providers to remain up to date on available technologies and to evaluate their scientific and clinical validity.

Users can document menstrual cycle data in a variety of ways by utilizing either qualitative or quantitative signs and symptoms of fertility. The three major categories of tracking technologies include smartphone applications (apps), temperature tracking devices, and at-home urine hormone tests. There are now over one thousand smartphone apps to track the menstrual cycle, but multiple studies have demonstrated that many of these apps are inaccurate at pinpointing the user’s fertile window [4,5,6]. Inaccurate prediction of the fertile window in the menstrual cycle has consequences for both users trying to achieve and those trying to avoid pregnancy. A growing interest in personalized menstrual cycle tracking has fueled the demand for smartphone apps, yet many of the available apps are unlikely to significantly improve users’ fertility and menstrual health literacy [6].

Another category of tracking technologies is temperature tracking devices. Recent innovations in temperature tracking technology have made it easier to track temperature changes across the menstrual cycle, controlling for the effects of changes in sleep duration, movement, and time of day. A few temperature devices marketed for menstrual cycle tracking include Ava, Tempdrop™, and Oura [7,8]. Several menstrual cycle apps, such as Read Your Body [9] and Natural Cycles [10], integrate temperature tracking with the aim of providing a more precise and seamless user experience.

In addition to smartphone apps and temperature tracking devices, there has been significant growth in the development of at-home urine hormone testing devices that can measure multiple reproductive hormone levels across the menstrual cycle. Direct-to-consumer urine hormone testing devices include, but are not limited to, the Clearblue^®^ Fertility Monitor [11], Proov^®^ [12], Inito Fertility Monitor [13], Mira^®^ Fertility Tracker [14], and Oova™ Fertility Tracker [15]. Prior to these technologies, it was neither feasible nor cost-effective to measure hormone levels at multiple timepoints throughout the menstrual cycle. These technologies are in the early stages of clinical validation, and there is an urgent need for research to support the use of urine hormone testing to predict ovulation.

These tracking methods are marketed for various purposes, most notably to help women identify the fertile window to achieve or avoid pregnancy. Reproductive disorders, such as polycystic ovary syndrome (PCOS), endometriosis, and infertility, affect 10-15% of reproductive-aged women [16,17] and are characterized by irregular menstrual cycles and abnormal hormonal patterns. However, these tracking methods have not been evaluated for women with irregular menstrual cycles. Given the potential for these technologies to screen for and guide the management of common reproductive disorders, it is imperative that they be validated for use in these populations.

An understanding of the current landscape of menstrual tracking technology use is necessary in order to perform targeted and patient-oriented research in this area. This information will provide the foundation for further validation studies of these technologies for specific reproductive health needs. Therefore, the purpose of this study was to examine the current menstrual technology landscape and explore how these technologies are being used within the context of common hormonal and reproductive disorders, like PCOS, endometriosis, and infertility.

## 2. Materials and Methods

### 2.1. Design

This study was a cross-sectional survey of menstrual cycle tracking technology use. Participants were recruited in January–March 2023 using social media groups and an email listserv. The primary source of recruitment was Facebook groups with an emphasis on fertility awareness-based methods, menstrual cycle tracking, natural family planning, PCOS, and infertility. When required, permission was sought from Facebook group administrators before posting study information. Study recruitment information was also sent to a specific group of menstrual cycle educators (Marquette Method, nfp.marquette.edu) via an email listserv, and instructors were asked to share the study information with their clients since this method specifically integrates urine hormone tracking.

Inclusion criteria were: (1) female, (2) between the ages of 18 and 50, (3) English-speaking, (4) access to a computer, and (5) use of one of the following tracking technologies: smartphone application, at-home urine hormone test or monitor, and temperature tracking device. Exclusion criteria were: (1) male, (2) younger than 18 years old or older than 50 years old, and (3) non-English speaking. All participants provided written consent at enrollment. The only personal identifiable information collected was participant emails, and only if they chose to participate in a drawing to receive one of ten $20 Amazon gift cards. Once completed, email addresses were deleted and not associated in any way with the study data. The study was approved by the institutional review board at Long Island University (project ID number: 23/01-001).

### 2.2. Data Collection

Data were collected from participants using an electronic survey with Qualtrics (see Supplementary Materials). We collected information on participant demographics as well as menstrual cycle characteristics, reproductive health history, and menstrual cycle tracking behavior. Participants reported their age, race and ethnicity, marital status, education, household income, employment, and religion. Menstrual cycle characteristics collected were menstrual cycle length and regularity. Participants provided self-reported diagnoses of the following reproductive disorders: PCOS, endometriosis, >6 months to achieve pregnancy, and infertility. Questions regarding menstrual cycle tracking behavior included primary motivation for tracking, length of cycle tracking duration, type of tracking used, fertility awareness-based method (FABM) use, contribution of tracking technology to reproductive disorder diagnosis, satisfaction of use, and contribution to reproductive health knowledge.

### 2.3. Statistical Analyses

We obtained means and standard deviations or frequencies and percentages as appropriate to describe characteristics of the sample. All statistical analyses were conducted using IBM Statistical Package for Social Science software (SPSS Version 28.0) [18].

## 3. Results

### 3.1. Participant Characteristics

**Figure 1:**
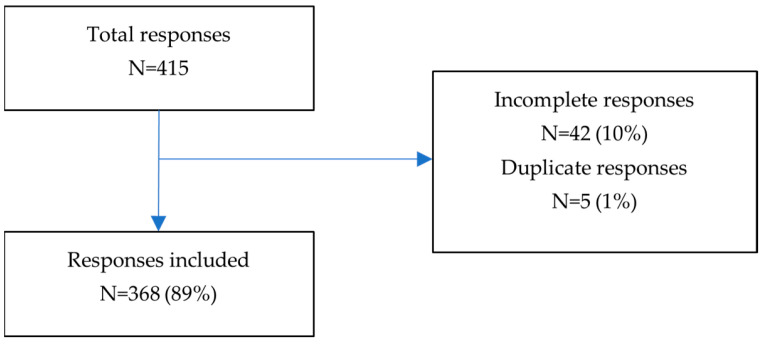
Flowchart describing sample size.

A total of 415 women responded to the survey. After removing incomplete responses (*n* = 42, 10%) and duplicates (*n* = 5, 1%), 368 (89%) participants were included in the analysis (Figure 1).

The mean age of the participants was 31.2 (range 20–49, SD 5.56). The majority were white (92.9%), married (91.6%), Christian (89.4%), and had at least a bachelor’s degree (86.2%) (Table 1).

### 3.2. Menstrual Cycle Characteristics and Reproductive Health History

The majority of study participants (*n* = 325, 88.3%) reported a menstrual cycle length between 25 and 35 days. Sixteen (4.3%) reported a menstrual cycle length < 25 days, nineteen (5.2%) reported a menstrual cycle length > 35 days, and six (1.6%) were unsure. When asked about menstrual cycle regularity, 282 (76.6%) reported a regular menstrual cycle, 76 (20.7%) reported an irregular menstrual cycle, and 4 (1.1%) reported that their menstrual cycle stopped (e.g., menopause). Within self-reported diagnoses of reproductive disorders, 55 (14.9%) reported PCOS, 35 (9.5%) reported endometriosis, 56 (15.2%) reported ever having tried for 6 months to get pregnant without becoming pregnant, and 20 (5.4%) reported infertility (Table 2).

### 3.3. Menstrual Cycle Tracking Motivation and Duration

Women had various motivations for tracking their menstrual cycles. The majority of women (*n* = 268, 72.8%) selected “to avoid getting pregnant” as the primary motivation, followed by “to learn more about my reproductive health” (*n* = 43, 11.7%), “to help me get pregnant” (*n* = 21, 5.8%), and “to track symptoms” (*n* = 18, 4.9%). Duration of menstrual tracking ranged from less than six months to over ten years. The majority of participants (*n* = 333, 91.5%) in this study have been tracking for more than a year, and 250 (67.9%) participants have been tracking for four years or more.

### 3.4. Utilization of Fertility Awareness-Based Methods (FABMs)

Three hundred and fifty-six (96.7%) participants reported using a FABM to track and interpret their menstrual cycle data. The Marquette Method [19], which utilizes urine hormone tracking, was the most frequently used method (*n* = 274, 68.2%), followed by the Creighton Model FertilityCare^TM^ System, which utilizes cervical mucus monitoring [20] (*n* = 53, 13.2%), the Billings Ovulation Method^®^, which also utilizes cervical mucus monitoring [21] (*n* = 28, 7%), the Symptothermal Method, which utilizes temperature tracking and cervical mucus monitoring [22] (*n* = 30, 7.5%), and FEMM, which utilizes hormonal and other indicators of menstrual health [23] (*n* = 11, 2.7%).

### 3.5. Reported Types of Menstrual Cycle Tracking Technology

Participants were asked to indicate which menstrual cycle tracking technologies they currently use, with the option to select more than one. The most frequently used technology was a urine hormone test or a monitor (*n* = 299, 81.3%), followed by a smartphone app (*n* = 253, 68.8%), and a temperature tracking device (*n* = 116, 31.5%).

Two hundred and ninety-nine women (81.3%) used a urine hormone test or device to track their menstrual cycle. The most frequently used urine hormone testing device was the Clearblue^®^ Fertility Monitor (49.3%), followed by luteinizing hormone (LH) test strips (34.1%), and PROOV progesterone test strips (12.2%). Newer quantitative technologies were much less frequently used, likely because they have not yet been significantly established in the market. Only 19 (3.6%) reported using the Mira Fertility Tracker and 3 (0.56%) reported using the Inito Fertility; none had used the Oova Fertility Tracker.

**Figure 2:**
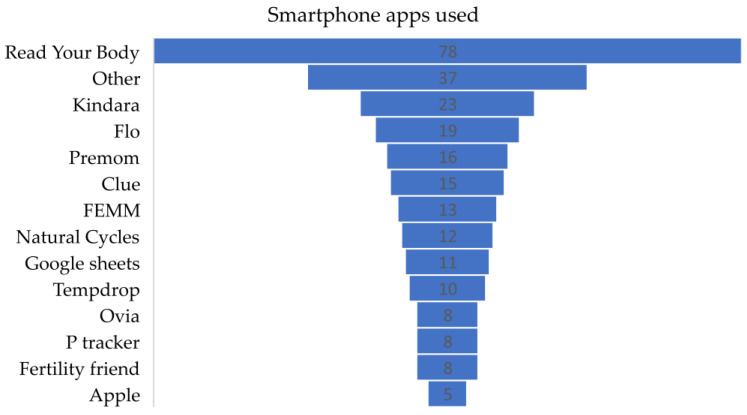
Frequency of smartphone apps used.

Two hundred and fifty-three women (68.8%) women used a smartphone app to track their menstrual cycle. Participants using a smartphone app to track their menstrual cycle identified 34 different menstrual cycle tracking apps. The five most frequently reported apps were Read Your Body, Kindara, Flo, Premom, and Clue. Apps used by at least five participants are presented in Figure 2. Apps used by fewer than five participants are include in Figure 2 in the “other” category.

The most frequently tracked parameters were “when your period starts” (28.9%), “sexual activity” (21.3%), and “urine hormone test results” (21.3%), followed by “daily symptoms” (16.4%), “temperature” (5.9%), and “cervical mucus” (6.3%). When asked about other parameters that were not listed as options in the survey, responses included: exercise, mood symptoms, period symptoms, medications, energy level, activity, photos, fertile window, and peak day.

Participants were able to select more than one reason for tracking using a smartphone app. The most common reason for tracking using an app was “to determine which days of the month are fertile” (40.3%) followed by “to track symptoms” (27%), “to provide reproductive health education” (18.6%), and “to help manage a health condition” (7.8%).

One hundred and sixteen (31.5%) women used a temperature tracking device to track their menstrual cycle. The most frequently used temperature tracking device was Tempdrop™ (46.7%) followed by over-the-counter basal body thermometers (37.2%).

### 3.6. Utilization of Menstrual Cycle Tracking Technologies in Reproductive Disorder Diagnosis and User Satisfaction

**Figure 3:**
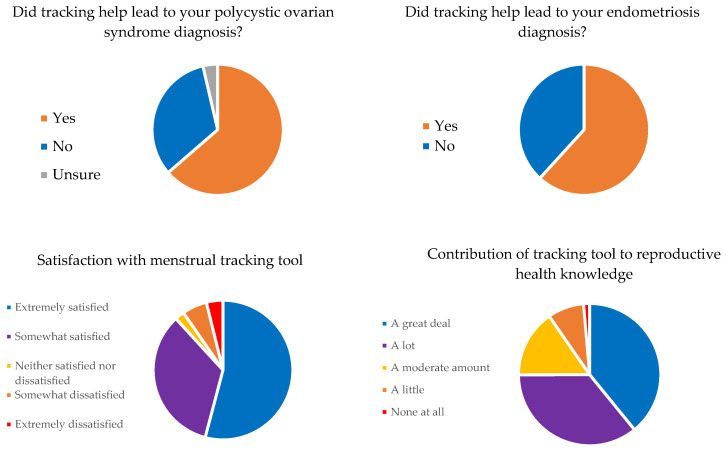
Effect of menstrual cycle tracking technology on user experience, satisfaction, and diagnoses.

The majority of women with endometriosis (61.8%), PCOS (63.6%), and infertility (75%) reported that utilizing menstrual tracking technologies helped lead to their reproductive disorder diagnoses (Figure 3).

The majority of participants reported being either “extremely satisfied” (53.5%) or “somewhat satisfied” (33.7%) with the technologies they were using. And, when asked about the contribution of tracking technologies to their reproductive health knowledge, 38.6% responded that menstrual tracking technologies contributed “a great deal” and 35.3% responded that tracking technologies contributed “a lot” (Figure 3).

## 4. Discussion

This study represents menstrual cycle technology use in a sample of women with significant experience tracking their cycles (67.9% tracking four years or more) who provided important insights into this evolving area of women’s health. The majority of the women in our study reported avoiding pregnancy as their primary motivation for using these technologies. The birth rate in the United States is 1.64 and an increasing percentage of women are giving birth at age 35 or older, so women spend the majority of their reproductive years avoiding pregnancy [24]. Consequently, it is not surprising that most respondents are trying to avoid a pregnancy. The only standalone technology validated for avoiding pregnancy is Natural Cycles [25], although the Marquette Method of Natural Family Planning, which incorporates multiple technologies, has also been validated for avoiding pregnancy [16,25]. Among FABMs, this was the most commonly used method by participants in our survey likely due to the study recruitment methods. To validate other menstrual cycle technologies for avoiding pregnancy, research is needed to evaluate the ability of these technologies to accurately predict the fertile window and to properly assess survival analysis for avoiding pregnancy. Prior research has demonstrated that many period tracker apps provide conflicting information on an ovulation day and a fertile window [4,6,26]. Several consumer-available tracking technologies are in the early stages of reliability testing for tracking or confirming ovulation [7,13,27,28]. More research is needed to validate their use for family planning.

Urine hormone testing was the most commonly utilized category of tracking technologies in our sample. The most likely explanation for this is that 68.2% of the women in this study reported using the Marquette Method. The Marquette Method is a FABM that combines threshold-based urine hormone test results from the Clearblue fertility monitor with a method-specific algorithm to help women identify their fertile window [19,29]. There are other studies evaluating the use of menstrual tracking technologies in women trying to conceive. In a population of 5,688 women trying to conceive, Stanford et al. (2019) found that the most common fertility indicator was charting menstrual cycle days [30]. And, in a recent study of 955 women participating in the Nurses’ Health Study, the three most tracked fertility indicators among women trying to conceive were menstrual cycle tracking, ovulation prediction kits, and cervical mucus monitoring [31]. The findings in this study may differ from prior studies due to the number of women using tracking technologies to avoid pregnancy in our study as well as the number of women using the Marquette Method, a urine hormone-based FABM.

Another finding from our study was that many women use more than one technology to track their menstrual cycle. Possible explanations for this are the inability of any one technology to achieve the users’ primary motivations for tracking and the need for greater integration across available technologies. In order to increase efficiency in menstrual cycle tracking, there is a need for apps with the ability to integrate temperature and/or urine hormone tracking [4]. Another explanation for this finding is that users’ needs and preferences are not a one-size-fits-all scenario. There is need for multiple and diverse tracking technologies to meet the varied needs of users. For instance, there are significant differences between apps that act as an electronic chart—where users input and interpret their own observations (e.g., Read Your Body)—and apps that provide interpretations of the users’ data based on algorithms (e.g., Natural Cycles). An additional consideration is the users’ perception of data security, which may be a result of recent data breaches from a few major menstrual cycle tracking apps [32]. There is a need to address the security and privacy of this data, so that women can approach these technologies with confidence and to ensure that these technologies align with the users’ best interests [33].

In this study, we also explored the use of menstrual cycle tracking technology by women with common reproductive disorders, including PCOS, endometriosis, and infertility. Many of these are designed for women with normal menstrual cycles, limiting their application to women with irregular menstrual cycles. Irregular and long menstrual cycles are common among women of reproductive age and are associated with greater risk for chronic cardiovascular and metabolic diseases [34,35]. These technologies play an important role in helping women identify menstrual cycle irregularities and may prompt women to seek an evaluation by a healthcare provider. The majority of women with self-reported diagnoses of PCOS and endometriosis in our study reported that the use of tracking technologies aided in the diagnosis (62–75%). As diagnosis of these conditions is frequently delayed [36,37], tools that have the potential to shorten the time to diagnosis could have a considerable impact on treatment outcomes for women with these conditions. These tracking technologies have tremendous clinical potential to assist in the early detection of common reproductive disorders. While research in this area is limited, Jain et al. (2021) found that data from Flo app users could be utilized to improve the phenotypic characterization of PCOS [38], and in another study, Rodriguez et al. (2020) pilot tested a predictive model that generated a PCOS risk score in the menstrual tracking app, Clue [39].

The findings of our study need to be interpreted within the context of several limitations. Our study sample contains a relatively high proportion of participants who were Christian, college graduates, employed, white, and married. The results may not be generalizable to women of different demographic, geographic, ethnic, or socioeconomic backgrounds. Future studies would benefit from exploring use in a more diverse population. We also acknowledge potential bias due to the high percentage of Marquette Method users in our population, which may overrepresent those using urine hormone testing to track their menstrual cycles.

## 5. Conclusions

The recent advances in menstrual tracking technologies sparked by increased consumer demand for reliable tools to track menstrual cycle health data highlight the need to validate these tools to improve women’s reproductive health care. As women are not routinely screened for common reproductive disorders, menstrual tracking technologies may be able to bridge the gap, serving as screening tools to improve the clinical detection and management of these conditions.

## Figures and Tables

**Table 1:** Demographic characteristics.

	*n*	%
Education		
Some High School	5	1.4
High School	23	6.3
Associate’s Degree	23	6.3
Bachelor’s Degree	182	49.5
Master’s Degree	106	28.8
Doctorate	29	7.9
Racial/ethnic background *		
American Indian or Alaskan	3	0.8
Asian	6	1.6
Black or African American	5	1.4
Hispanic or Latino	35	9.5
Other	4	1.1
Prefer not to say	8	2.2
White	342	92.9
Marital status		
Married	337	91.8
Never married	30	8.2
Religion		
Agnostic	3	0.8
Atheist	5	1.4
Jewish	1	0.3
Mormon	1	0.3
Nothing in particular	21	5.7
Orthodox (Greek or Russian Orthodox)	3	0.8
Other	5	1.4
Protestant	29	7.9
Roman Catholic	300	81.5
Employment *		
Employed full time	166	43.3
Employed part time	104	27.2
Unemployed	81	21.1
Student	25	6.5
Prefer not to say	7	1.8
Total household income		
Less than $50,000	27	7.3
$50,000–$74,999	52	14.1
$75,000–$99,999	72	19.6
$100,000–$124,999	78	21.2
$125,000–$149,999	45	12.2
More than $150,000	82	22.3
Prefer not to say	11	3.0

* Could select more than one.

**Table 2:** Menstrual Cycle Characteristics and Reproductive Health History.

	*n*	%
Menstrual Cycle Length		
<25 days	16	4.3
25-35 days	325	88.3
>35 days	19	5.2
Unsure	6	1.6
Menstrual Cycle Regularity		
Regular	282	76.6
Irregular	76	20.7
Stopped	4	1.1
Polycystic Ovary Syndrome		
Yes	55	14.9
No	205	82.9
Unsure	7	1.9
Endometriosis		
Yes	35	9.5
No	326	88.6
Unsure	7	1.9
Tried for >6 months		
Yes	56	15.2
No	307	83.4
Unsure	4	1.1
Infertility		
Yes	20	5.4
No	342	92.9
Unsure	5	1.4

## Data Availability

Not applicable.

## Supplementary Materials

The following supporting information can be downloaded at: https://www.mdpi.com/article/10.3390/medicina59091509/s1, Survey questionnaire.
