# Supplementary material for: Survey Analysis of Quantitative and Qualitative Menstrual Cycle Tracking Technologies
Source: Medicina (Kaunas). 2023 Aug 22;59(9):1509. doi: 10.3390/medicina59091509 (PMC10534579; doi:10.3390/medicina59091509)
Supplement: Supplementary file 1 [file medicina-59-01509-s001.zip › medicina-2533051-supplementary.pdf]

Cycle Tracking Technology Survey

Survey Flow

Block: Default Question Block (11 Questions)
Standard: Block 4 (27 Questions)
Standard: Block 1 (11 Questions)
Standard: Block 2 (19 Questions)
Standard: Block 3 (22 Questions)
Standard: Block 5 (1 Question)

EndSurvey:

Page Break

Q1 What is your age?

▼ (1) ... >50 (35)

Skip To: End of Survey If What is your age? =

Skip To: End of Survey If What is your age? = >50

Q4 What sex were you assigned at birth?

○ Male (1)

○ Female (2)

Skip To: End of Survey If What sex were you assigned at birth? = Male

Q2 What is your race?

○ White (1)

○ Black or African American (2)

○ American Indian or Alaska Native (3)

○ Asian (4)

○ Native Hawaiian or Other Pacific Islander (5)

○ Some other race (6)

○ Prefer not to say (7)

Q8 What is your ethnicity?

- ○ Hispanic or Latino (1)
- ○ Not Hispanic or Latino (2)
- ○ Prefer not to say (3)

Q3 What is the highest degree or level of education you have completed?

- ○ Some High School (1)
- ○ High School (2)
- ○ Associate's Degree (3)
- ○ Bachelor's Degree (4)
- ○ Master's Degree (5)
- ○ Doctorate (6)
- ○ Prefer not to say (7)

Q5 What is your best estimate of the current total yearly income of all individuals living in your household (for example, family members) with whom you share finances?

- Less than $50,000 (1)
- $50,000 - $74,999 (2)
- $75,000 - $99,999 (3)
- $100,000 - $124,999 (4)
- $125,000 - $149,999 (5)
- More than $150,000 (6)
- Prefer not to say (7)

Q6 Describe your employment. (Select all that apply)

- Employed full time (1)
- Employed part time (2)
- Unemployed (3)
- Retired (4)
- Student (5)
- Disabled (6)
- Prefer not to say (7)

Q7 What is your marital status?

- ◯ Married (1)
- ◯ Widowed (2)
- ◯ Divorced (3)
- ◯ Separated (4)
- ◯ Never married (5)
- ◯ Prefer not to say (6)

Q11 What is your present religion, if any?

- ◯ Protestant (1)
- ◯ Roman Catholic (2)
- ◯ Mormon (3)
- ◯ Orthodox, such as Greek or Russian Orthodox (4)
- ◯ Jewish (5)
- ◯ Muslim (6)
- ◯ Buddhist (7)
- ◯ Hindu (8)
- ◯ Atheist (9)
- ◯ Agnostic (10)
- ◯ Other (11)
- ◯ Nothing in particular (12)

End of Block: Default Question Block

Start of Block: Block 4

Q91 The next set of questions assess your knowledge of female fertility and the menstrual cycle. The answer choices are true, false, or don’t know.

Q62 An ovulation is the releasing of an egg from the ovary.

- True (1)
- False (2)
- Don't know (3)

Q63 There are about 6 days in each menstrual cycle when a woman is able to get pregnant.

- True (1)
- False (2)
- Don't know (3)

Q64 The egg that a woman releases from her ovary lives for 12 to 24 hours if it is not fertilized.

- True (1)
- False (2)
- Don't know (3)

Q65 The length of a menstrual cycle refers to the first day of the period until the day before the next period.

- True (1)
- False (2)
- Don't know (3)

Q66 Normal menstrual cycle length ranges between 21 to 35 days.

- True (1)
- False (2)
- Don't know (3)

Q67 Sperm from a man can live up to 5 days in a woman’s body with good cervical mucus.

- True (1)
- False (2)
- Don't know (3)

Q68 Ovulation always occurs on the 14th day of each menstrual cycle.

- True (1)
- False (2)
- Don't know (3)

Q69 A woman is born with all the eggs she will ever have in her life.

- True (1)
- False (2)
- Don't know (3)

Q70 A woman’s age is one of the strongest risk factors for infertility.

- True (1)
- False (2)
- Don't know (3)

Q71 Female fertility remains stable from puberty until menopause.

- True (1)
- False (2)
- Don't know (3)

Q72 Sexually transmitted infections increase the risk of infertility.

- True (1)
- False (2)
- Don't know (3)

Q73 The quality and quantity of a woman’s egg decline as she gets older.

- True (1)
- False (2)
- Don't know (3)

Q74 Women remain fertile even after menopause.

- True (1)
- False (2)
- Don't know (3)

Q75 A woman’s body weight may affect her chances of getting pregnant.

- True (1)
- False (2)
- Don't know (3)

Q76 The likelihood of conceiving varies with a woman’s age.

- True (1)
- False (2)
- Don't know (3)

Q77 The risk of having a baby with Down syndrome increases with a woman’s age.

- True (1)
- False (2)
- Don't know (3)

Q78 Aging may increase a woman’s chance of miscarriage.

- True (1)
- False (2)
- Don't know (3)

Q79 A woman is most fertile in her 30s.

- True (1)
- False (2)
- Don't know (3)

Q80 Smoking decreases a woman’s fertility.

- True (1)
- False (2)
- Don't know (3)

Q81 Being overweight may decrease a woman’s chance of getting pregnant.

- True (1)
- False (2)
- Don't know (3)

Q82 Being underweight may increase a woman’s chance of getting pregnant.

- True (1)
- False (2)
- Don't know (3)

Q83 Regular use of marijuana has no impact on a woman’s ability to get pregnant.

- True (1)
- False (2)
- Don't know (3)

Q84 Drinking more than 7 cups of caffeinated beverages a day lowers a woman’s chance of getting pregnant.

- True (1)
- False (2)
- Don't know (3)

Q85 The timing of ovulation may vary in each menstrual cycle.

- True (1)
- False (2)
- Don't know (3)

Q86 A woman over 35 years old should seek medical help if she cannot get pregnant after 6 months of trying to get pregnant.

- True (1)
- False (2)
- Don't know (3)

Q87 Cervical mucus is an indicator of changes in female fertility during the menstrual cycle.

- True (1)
- False (2)
- Don't know (3)

End of Block: Block 4

Start of Block: Block 1

Q93 The next set of questions is about your overall health and reproductive health.

Q9 What is your current height?

○ Feet (1) ____

○ Inches (2) ____

Q10 What is your current weight?

○ Pounds (1) ____

Q12 At what age did you have your first menstrual period?

▼ 7 years old or younger (1) ... I have never had a period (12)

Q13 A menstrual period is regular when you can usually predict about when your next period will start. Would you say that your period is usually regular?

○ Yes (1)

○ No, they have never been regular (2)

○ No, they have been irregular for a few months (3)

○ No, my periods have stopped (4)

Q14 Menstrual cycle length is the number of days from the first day of one menstrual period to the first day of your next period. Normal menstrual cycle lengths range from 21 to 35 days. What is your normal menstrual cycle length?

▼ 21 days (1) ... I'm not sure (17)

Q15 Have you had your menstrual period in the last 12 months?

- Yes (1)
- No (2)
- I don't remember (3)

Display This Question:

If Have you had your menstrual period in the last 12 months? = Yes

Q20 How many periods have you had in the last 12 months?

▼ 1 (1) ... >15 (16)

Display This Question:

If Have you had your menstrual period in the last 12 months? = Yes

Q17 Is your menstrual cycle often (more than twice a year) more than 35 days?

- Yes (1)
- No (2)
- I'm not sure (3)

Display This Question:

If Have you had your menstrual period in the last 12 months? = Yes

Q18 Has your menstrual period been irregular over the last 12 months?

- Yes (1)
- No (2)
- I'm not sure (3)

Q21 Do you (or did you) usually experience the following symptoms the days before or around your menstrual periods? (Select all that apply)

- Anger or irritability (1)
- Anxiety or tension (2)
- Tearfulness or increased sensitivity to rejection (3)
- Feeling depressed or hopeless (4)
- Difficulty with sleeping (5)
- Abdominal pain (so that you need to take pain killers) (6)
- Breast tenderness, abdominal bloating and/or swelling (7)
- Headache (8)
- None (9)

End of Block: Block 1

Start of Block: Block 2

Q26 Has a doctor or health professional ever told you that you have polycystic ovarian syndrome (PCOS)?

◯ Yes (1)

◯ No (2)

◯ Unsure (3)

Display This Question:

If Has a doctor or health professional ever told you that you have polycystic ovarian syndrome (PCOS)? = Yes

Q27 How old were you when you first had symptoms of polycystic ovarian syndrome (PCOS)?

▼ 10 (1) ... >45 (37)

Display This Question:

If Has a doctor or health professional ever told you that you have polycystic ovarian syndrome (PCOS)? = Yes

Q28 How old were you when a doctor told you that you had polycystic ovarian syndrome (PCOS)?

▼ 10 (1) ... >45 (37)

Q35 Did/do you experience any of the following symptoms of polycystic ovarian syndrome (PCOS)? (Select all that apply)

☐ Acne (1)

☐ Excessive hair growth (2)

☐ Weight gain (3)

☐ Irregular menstrual cycle (4)

☐ Cysts in ovaries (5)

☐ High testosterone/androgen levels (6)

☐ Other, please describe (7) ____

Q28 Has a doctor or health professional ever told you that you have endometriosis?

○ Yes (1)

○ No (2)

○ Unsure (3)

Display This Question:

If Has a doctor or health professional ever told you that you have endometriosis? = Yes

Q29 How old were you when you first had symptoms of endometriosis?

▼ 10 (1) ... >45 (37)

Display This Question:

If Has a doctor or health professional ever told you that you have endometriosis? = Yes

Q30 How old were you when a doctor told you that you had endometriosis?

▼ 10 (1) ... >45 (37)

Q36 Did/do you experience any of the following symptoms of endometriosis? (Select all that apply)

- ▢ Painful periods (1)
- ▢ Pain during sex (2)
- ▢ Pain with bowel movements (3)
- ▢ Heavy menstrual bleeding (4)
- ▢ Infertility (5)
- ▢ Fatigue (6)
- ▢ Bloating, nausea, diarrhea (7)
- ▢ Other, please describe (8) ____

Q30 Have you ever tried for six months or more to become pregnant without becoming pregnant?

- ○ Yes (1)
- ○ No (2)
- ○ Unsure (3)

Display This Question:

If Have you ever tried for six months or more to become pregnant without becoming pregnant? = Yes

Q31 Thinking about the first time you experienced this, how old were you?

▼ 16 (1) ... 50 (35)

Display This Question:

If Have you ever tried for six months or more to become pregnant without becoming pregnant? = Yes

Q32 How long did you try for?

- 6-12 months (1)
- 1 year or more (2)

Q33 Has a doctor or health professional ever diagnosed you with infertility?

- Yes (1)
- No (2)
- Unsure (3)

Display This Question:

If Has a doctor or health professional ever diagnosed you with infertility? = Yes

Q34 How old were you when a doctor told you that you had infertility?

▼ 16 (1) ... 50 (35)

Display This Question:

If Has a doctor or health professional ever diagnosed you with infertility? = Yes

Q38 Are you currently being treated for infertility?

- ○ Yes (1)
- ○ No (2)

Q37 Do you currently using any of the following for contraception? (Select all that apply)

- ☐ Pill (1)
- ☐ Patch (2)
- ☐ Vaginal ring (3)
- ☐ Injection (4)
- ☐ Patch (5)
- ☐ Hormonal IUD (6)
- ☐ Non-hormonal IUD (7)
- ☐ Condoms (8)
- ☐ Other, please describe (9) ____

Q40 Are you currently taking hormonal contraceptives?

- ○ Yes (1)
- ○ No (2)

Display This Question:

If Are you currently taking hormonal contraceptives? = Yes

Q42 How long have you been taking hormonal contraceptives? (If you have stopped and restarted, please estimate the total time you have used contraceptives)

- ○ Less than 6 months (1)
- ○ 6 months-1 years (2)
- ○ 1-2 years (3)
- ○ 2-3 years (4)
- ○ 3-4 years (5)
- ○ 4-5 years (6)
- ○ More than 5 years (7)

Display This Question:

If Are you currently taking hormonal contraceptives? = Yes

Q41 For which of the following indications do you use a hormonal contraceptive? (Select all that apply)

- ☐ Contraception (1)
- ☐ Irregular periods (2)
- ☐ Painful periods (3)
- ☐ Heavy menstrual bleeding (4)
- ☐ Polycystic ovarian syndrome (5)
- ☐ Acne (6)
- ☐ Endometriosis (7)
- ☐ Other, please describe (8) ____

Q39 Are you currently being treated for menopausal symptoms (i.e., hormonal replacement therapy)?

- ○ Yes (1)
- ○ No (2)

End of Block: Block 2

Start of Block: Block 3

Q94 This final set of questions is about your menstrual cycle tracking behavior.

Q43 Did you or do you track your menstrual cycle (i.e., use a phone application, monitor your hormone levels, track your temperature)?

- Yes (1)
- No (2)

Display This Question:

If Did you or do you track your menstrual cycle (i.e., use a phone application, monitor your hormone... = Yes

Q52 Which of the following describes your primary motivation for tracking your menstrual cycle? (Select the primary reason)

- To learn more about my reproductive health (1)
- To help me get pregnant (2)
- To avoid getting pregnant (3)
- To track symptoms (4)
- Other, please describe (5) ____

Display This Question:

If Did you or do you track your menstrual cycle (i.e., use a phone application, monitor your hormone... = Yes

Q95 How old were you when you started tracking your menstrual cycle?

▼ 16 years old or younger (1) ... 45 years old or older (31)

Display This Question:

If Did you or do you track your menstrual cycle (i.e., use a phone application, monitor your hormone... = Yes

Q96 How long have you been tracking your menstrual cycle?

- ◯ 6 months or less (1)
- ◯ 6 months-1 year (2)
- ◯ 1-3 years (3)
- ◯ 4-7 years (4)
- ◯ 7-9 years (5)
- ◯ 10 years or more (6)

Display This Question:

If Did you or do you track your menstrual cycle (i.e., use a phone application, monitor your hormone... = Yes

Q44 Which of the following do you use to track your menstrual cycle? (Select all that apply)

- ▢ Phone application (app) (1)
- ▢ Urine hormone test or monitor (2)
- ▢ Temperature (3)
- ▢ Other, please describe (4) ____

Display This Question:

If Which of the following do you use to track your menstrual cycle? (Select all that apply) = Phone application (app)

Q45 Which of the following phone applications (apps) do you PRIMARILY use to track your menstrual cycle?

- ○ Flo (1)
- ○ Clue (2)
- ○ Natural Cycles (3)
- ○ Kindara (4)
- ○ Other, please describe (5) ____

Display This Question:

If Which of the following do you use to track your menstrual cycle? (Select all that apply) = Phone application (app)

Q46 Which of the following do you track in the app? (Select all that apply)

- ▢ When your period starts (1)
- ▢ Sexual activity (2)
- ▢ Daily symptoms (3)
- ▢ Urine hormone test results (4)
- ▢ Other, please describe (5) ____

Display This Question:

If Which of the following do you use to track your menstrual cycle? (Select all that apply) = Phone application (app)

Q47 Which of the following reasons do you track your menstrual cycle using an app? (Select all that apply)

- ☐ To determine which days of the month are fertile (1)
- ☐ To track symptoms (2)
- ☐ To provide reproductive health education (3)
- ☐ To help manage a health condition (4)
- ☐ Other, please describe (5) ____

Display This Question:

If Which of the following do you use to track your menstrual cycle? (Select all that apply) = Urine hormone test or monitor

Q48 Which of the following urine hormone tests and/or monitors do you currently use to track your menstrual cycle? (Select all that apply)

- ☐ Luteinizing hormone (LH) test strips (1)
- ☐ PROOV progesterone test strips (2)
- ☐ Clearblue Fertility Monitor (3)
- ☐ Mira Fertility Tracker (4)
- ☐ Inito Fertility Monitor (5)
- ☐ Oova Fertility Translator (6)
- ☐ Other, please describe (7)

Display This Question:

If Which of the following do you use to track your menstrual cycle? (Select all that apply) = Temperature

Q49 Which of the following temperature tracking devices do you currently use to track your menstrual cycle? (Select all that apply)

- ▢ Tempdrop (1)
- ▢ Ava (2)
- ▢ Oura (3)
- ▢ Over the counter basal body thermometer (4)
- ▢ Other, please describe (5) ____

Q50 Do you use a fertility awareness based method or natural family planning method?

- ○ Yes (1)
- ○ No (2)

Display This Question:

If Do you use a fertility awareness based method or natural family planning method? = Yes

Q51 Which fertility awareness based method do you use? (Select all that apply)

- ▢ Billings Ovulation Method (1)
- ▢ Creighton Model (2)
- ▢ Marquette Method (3)
- ▢ Other, please describe (4) ____

Display This Question:

If Has a doctor or health professional ever told you that you have polycystic ovarian syndrome (PCOS)? = Yes

Q53 Did tracking your menstrual cycle give you information that helped lead to your polycystic ovarian syndrome (PCOS) diagnosis?

- Yes (1)
- No (2)
- Unsure (3)

Display This Question:

If Did tracking your menstrual cycle give you information that helped lead to your polycystic ovaria... = Yes

Q54 Which of the following menstrual cycle tracking tools gave you the information that helped lead to your polycystic ovarian syndrome (PCOS) diagnosis?

- Phone app, please list (1) ____
- Urine hormone test or device, please list (2) ____
- Temperature tracking device, please list (3) ____
- Other, please list (4) ____

Display This Question:

If Has a doctor or health professional ever told you that you have endometriosis? = Yes

Q55 Did tracking your menstrual cycle give you information that helped lead to your endometriosis diagnosis?

- ○ Yes (1)
- ○ No (2)
- ○ Unsure (3)

Display This Question:

If Did tracking your menstrual cycle give you information that helped lead to your endometriosis dia... = Yes

Q57 Which of the following menstrual cycle tracking tools gave you the information that helped lead to your endometriosis diagnosis?

- ☐ Phone app, please list (1) ____
- ☐ Urine hormone test or device, please list (2) ____
- ☐ Temperature tracking device, please list (3) ____
- ☐ Other, please list (4) ____

Display This Question:

If Has a doctor or health professional ever diagnosed you with infertility? = Yes

Q56 Did tracking your menstrual cycle give you information that helped lead to your infertility diagnosis?

- ○ Yes (1)
- ○ No (2)
- ○ Unsure (3)

Display This Question:

If Did tracking your menstrual cycle give you information that helped lead to your infertility diagn... = Yes

Q58 Which of the following menstrual cycle tracking tools gave you the information that helped lead to your infertility diagnosis?

☐ Phone app, please list (1) ____

☐ Urine hormone test or device, please list (2) ____

☐ Temperature tracking device, please list (3) ____

☐ Other, please list (4) ____

Display This Question:

If Did you or do you track your menstrual cycle (i.e., use a phone application, monitor your hormone... = Yes

Q59 How satisfied are you with the menstrual cycle tracking tool you are using?

○ Extremely dissatisfied (1)

○ Somewhat dissatisfied (2)

○ Neither satisfied nor dissatisfied (3)

○ Somewhat satisfied (4)

○ Extremely satisfied (5)

Display This Question:

If Did you or do you track your menstrual cycle (i.e., use a phone application, monitor your hormone... = Yes

Q60 How much has the menstrual cycle tracking tool you are using helped you learn about your reproductive health?

- ◯ None at all (1)
- ◯ A little (2)
- ◯ A moderate amount (3)
- ◯ A lot (4)
- ◯ A great deal (5)

Q61 Please describe what improvements you would like to see in menstrual cycle tracking tools (i.e., additional information that would be helpful):

____

End of Block: Block 3

Start of Block: Block 5

Q97 If you would like to be entered into a drawing to receive one of ten $20 Amazon gift cards, please provide your email. Your email will not be linked with your data in any way and it will be deleted once the drawing is complete.

____

End of Block: Block 5
